# Engineered CAR-T extracellular vesicles loaded with miR-17-5p inhibitor suppress hepatocellular carcinoma progression

**DOI:** 10.3389/fimmu.2026.1919449

**Published:** 2026-09-16

**Authors:** Zhifeng Zhou, Huiling Wu, Shen Zhao, Mingshui Chen, Wansong Lin, Jieyu Li, Qingfeng Zheng

**Affiliations:** 1 Laboratory of Immuno-Oncology, Clinical Oncology School of Fujian Medical University, Fujian Cancer Hospital, Fuzhou, China; 2 The School of Basic Medical Sciences, Fujian Medical University, Fuzhou, China; 3 Department of Gastrointestinal Medical Oncology, Clinical Oncology School of Fujian Medical University, Fujian Cancer Hospital, Fuzhou, China; 4 Department of Thoracic Surgery, Clinical Oncology School of Fujian Medical University, Fujian Cancer Hospital, Fuzhou, China

**Keywords:** CAR-T cell-derived extracellular vesicles, hepatocellular carcinoma (HCC), miR-17-5p inhibitor, natural killer (NK) cells, zebrafish xenografts

## Abstract

**Background:**

Hepatocellular carcinoma (HCC) immune escape is co-driven by tumor malignant proliferation and an immunosuppressive tumor microenvironment, characterized by frequently impaired natural killer (NK) cell function. Previous research indicated that HCC cell-secreted extracellular vesicles (EVs) deliver miR-17-5p, inhibiting the RUNX1 pathway and downregulating the activating receptor NKG2D in NK cells, which ultimately results in NK cell dysfunction. Although chimeric antigen receptor (CAR)-T therapy has achieved remarkable efficacy in hematological malignancies, it encounters significant challenges in treating solid tumors, including HCC. Recent studies have confirmed that CAR-T cell–derived EVs (CAR-T EVs) retain CAR targeting specificity and carry cytotoxic effectors such as granzyme B and perforin, demonstrating independent antitumor potential.

**Objective:**

This study aimed to develop engineered CAR-T EVs loaded with a miR-17-5p inhibitor (EV-miR inhibitor) and evaluate their combined therapeutic efficacy against HCC through direct tumor cytotoxicity and restoration of NK cell function.

**Methods:**

This study constructed NKG2D-targeted third-generation CAR-T cells, isolated CAR-T EVs via ultracentrifugation, characterized EV properties using multiple assays, and generated engineered CAR-T EVs loaded with a miR-17-5p inhibitor (EV-miR inhibitor). The therapeutic effects of this system against HCC were verified both *in vitro* and in a zebrafish HCC xenograft model.

**Results:**

CAR-T EVs exhibited characteristic cup-shaped morphology and robust expression of canonical EV markers together with the engineered NKG2D extracellular domain. Results indicated that CAR-T EVs entered HCC cells and exhibited concentration-dependent cytotoxicity against HCC cells without affecting the viability of normal hepatocytes. The loading of the miR-17-5p inhibitor was efficient, and the procedure did not alter the morphology, particle size, or marker expression of CAR-T EVs. EV-miR inhibitor effectively restored NKG2D and RUNX1 expression in miR-17-5p-overexpressing NK cells, significantly enhancing NK cell cytotoxicity against HCC and promoting IFN-γ secretion *in vitro*. *In vivo* experiments further confirmed that EV-miR inhibitor treatment markedly enhanced the antitumor activity of NK cells, leading to significant tumor growth inhibition in zebrafish xenografts.

**Conclusions:**

In conclusion, this study demonstrates that NKG2D-targeted CAR-T EVs exert combined anti-HCC activities through direct tumor cytotoxicity and restoration of NK cell function, providing a novel immunotherapeutic strategy for HCC.

## Introduction

Hepatocellular carcinoma (HCC) ranks among the most frequently diagnosed malignancies globally, characterized by extremely high mortality rates and a steadily increasing incidence over recent decades (1). The liver, as an inherently tolerogenic organ, maintains systemic immune homeostasis while simultaneously providing a favorable pre-metastatic niche for tumor immune escape (2). The complex immunosuppressive tumor microenvironment (TME) limits the effectiveness of conventional therapies and serves as a central driver of the high recurrence and metastasis rates observed in HCC (3). Despite breakthroughs in emerging immunotherapies, including immune checkpoint blockade (ICB) and adoptive cell therapy (ACT), significant challenges remain in overcoming the physical barriers of solid tumors and reversing local immunosuppression (3–5). As the primary effectors of innate anti-tumor immune surveillance, natural killer (NK) cells can recognize malignant cells via activating receptors without prior sensitization. They exert direct cytotoxicity by releasing perforin and granzymes and regulate adaptive immune responses through cytokine secretion, playing an essential role in anti-HCC immunity (8–11). However, accumulating evidence indicates that NK cells in the tumor tissues and peripheral blood of patients with HCC are generally dysfunctional, characterized by the significant downregulation of activating receptors such as NKG2D, and gradually impaired cytotoxicity as tumor progression occurs. Additionally, the expression levels of activating receptors on NK cells significantly correlate negatively with HCC clinical stages. Thus, reversing NK cell dysfunction has emerged as a critical focus for remodeling anti-tumor immunity in HCC (6, 7).

Extracellular vesicle (EV)-mediated microRNA transport represents a key mechanism through which HCC cells remodel the immune microenvironment and induce immune escape. Various HCC cell-derived EVs carry inhibitory microRNAs that induce immune cell dysfunction and facilitate tumor progression (8–11). As a core member of the miR-17–92 cluster, miR-17-5p is widely involved in regulating biological processes such as tumor cell cycle, proliferation, and invasion, and it has been confirmed to play a pro-tumorigenic role in multiple malignancies, including colorectal cancer and prostate cancer. However, its specific role in modulating the HCC immune microenvironment remains to be thoroughly elucidated (12, 13). Previous studies demonstrated that the content of miR-17-5p in EVs derived from HCC tissues was significantly higher than that in matched paracancerous tissues; miR-17-5p secreted by HCC cells can be transported into NK cells, downregulating NKG2D expression by targeting and inhibiting the transcription factor RUNX1, ultimately diminishing the recognition and killing ability of NK cells against tumor cells. This finding reveals a novel immune escape mechanism of HCC via the *HCC EVs-miR-17-5p-RUNX1-NKG2D* axis, highlighting miR-17-5p as a potential target for restoring NK cell function (14). The miR-17-5p inhibitor is an antisense complementary single-stranded nucleic acid designed to target the mature sequence of miR-17-5p, effectively blocking its silencing effect on target genes through sequence-specific binding and restoring the expression of RUNX1 and NKG2D, thereby theoretically reversing miR-17-5p-mediated NK cell dysfunction (14, 15). However, naked nucleic acids are prone to degradation by nucleases *in vivo* and possess inherent limitations such as low cellular uptake efficiency and high off-target risks, underscoring the urgent need for the development of safe and efficient targeted delivery vectors.

Chimeric antigen receptor T (CAR-T) cell therapy has achieved landmark efficacy in hematological malignancies, providing survival hope for tens of thousands of patients worldwide. However, its application in solid tumors, such as HCC, faces significant challenges, including low tumor infiltration efficiency, TME-mediated immunosuppression, and severe adverse events like cytokine release syndrome (CRS) (16, 17). Currently, several CAR-T products targeting Glypican-3 (GPC3), NKG2D ligand (NKG2DL), and other HCC-specific targets have been developed (18, 19). Previous studies confirmed that NKG2D-targeted CAR-T can specifically recognize tumor cells, effectively addressing off-target concerns, yet the risk of inducing CRS remains significant (20). Recently, CAR-T cell–derived EVs (CAR-T EVs) have emerged as a novel cell-free anti-tumor therapeutic strategy, attracting extensive attention. CAR-T EVs not only retain the CAR-targeted structure present on the surface of parental cells, but are also naturally enriched with cytotoxic effectors such as granzyme B and perforin, enabling independent direct anti-tumor activity (21, 22). Compared to parental CAR-T cells, CAR-T EVs offer several unique advantages: first, as a cell-free nanopreparation, they exhibit lower immunogenicity, fundamentally avoiding severe toxic reactions such as CRS, and significantly enhancing safety; second, their nanoscale size facilitates easier penetration through the stromal barriers of solid tumors, allowing for improved infiltration and enrichment in tumor tissues; additionally, CAR-T EVs can function as natural nanocarriers for targeted synergistic therapy by loading nucleic acids and drugs, making them particularly suitable for the combined intervention requirements of HCC (23). These characteristics position CAR-T EVs as a promising therapeutic platform in HCC immunotherapy.

Based on this research background, this study proposes the following scientific hypothesis: utilizing NKG2D-targeted CAR-T EVs as a natural nano delivery platform, a strategy that combines endogenous and exogenous loading can achieve comprehensive anti-HCC activities through two functional pathways: direct tumor cell killing and reversing NK cell dysfunction. Endogenously, NKG2D CAR is expressed in CAR-T cells via lentiviral transduction and incorporated onto the surface of EVs secreted by CAR-T, enabling these EVs to retain the capability to target and recognize HCC cells while directly killing tumors with their inherent effector molecules. Exogenously, loading a miR-17-5p inhibitor into CAR-T EVs facilitates efficient delivery of the inhibitor to dysfunctional NK cells, thereby restoring NK cell anti-tumor function by blocking the miR-17-5p/RUNX1 axis.

## Materials and methods

### Cell lines

All cells were maintained at 37 °C in a humidified incubator with 5% CO_2_ unless otherwise specified. Human HCC cell lines, MHCC-97H (97H) and Huh-7, were cultured in high-glucose Dulbecco’s Modified Eagle Medium (DMEM) supplemented with 10% (v/v) fetal bovine serum (FBS) and 1% (v/v) penicillin-streptomycin (100 U/mL penicillin, 100 μg/mL streptomycin). The immortalized human normal hepatic cell line THLE-2 was cultured according to the manufacturer’s instructions in its specialized medium. All cell lines were authenticated through short tandem repeat (STR) profiling prior to experiments and were regularly screened for mycoplasma contamination to ensure negative status throughout the study.

### Expression of NKG2D on peripheral blood NK cells in patients with hepatocellular carcinoma

This study received approval from the Fujian Cancer Hospital Institutional Ethics Committee (Ethics Approval Number: K2023-323-01). All enrolled patients with HCC provided written informed consent, and those with concurrent autoimmune diseases or recent histories of immune interventions were excluded. Fasting EDTA-anticoagulated peripheral whole blood was collected from patients, transported at 4 °C, and processed within 2 hours of sampling. The whole blood was directly stained with CD3/CD56/NKG2D surface antibodies, and red blood cells were lysed prior to machine detection. NK cell subpopulations were defined by CD3^–^CD56^+^ gating, with the positive expression proportion and mean fluorescence intensity of surface NKG2D analyzed using isotype control to calibrate non-specific staining.

### Generation of NKG2D-targeted CAR-T cells

A third-generation recombinant lentiviral vector encoding a GFP-tagged NKG2D-targeted chimeric antigen receptor (CAR) was pre-constructed for this study, and activated primary human T cells were transduced following the protocol outlined below. After *in vitro* activation, healthy T cells were stained with Acridine Orange/Propidium Iodide (AO/PI) for viability assessment, and only cells with viability ≥ 90% were eligible for subsequent experimentation. Qualified T cells were collected, washed three times with Dulbecco’s Phosphate-Buffered Saline (DPBS), and resuspended in half the final volume of fresh KBM-581 medium supplemented with 200 IU/mL recombinant human interleukin-2 (IL-2), adjusting to the appropriate working concentration. Recombinant lentivirus carrying the human NKG2D CAR fusion sequence was added at a multiplicity of infection (MOI) of 20, along with Polybrene at the manufacturer’s recommended dose. The mixture was gently mixed and placed in a humidified incubator at 37 °C with 5% CO_2_ for incubation. After 2 hours, pre-warmed fresh medium was added to achieve the standard working volume of the culture container, and cells were returned to the incubator for continuous culture. GFP fluorescence intensity was recorded every 72 hours to monitor transduction efficiency; seven days post-transduction, the CAR positive rate was assessed by flow cytometry.

### NK cell culture and lentiviral transduction for miR-17-5p overexpression

This study involving human peripheral blood samples was approved by the Institutional Review Board (Approval Number: K2025-053-01), and written informed consent was obtained from all healthy volunteers prior to sample collection. Briefly, 10 mL of peripheral venous blood was collected from each volunteer using heparin-coated blood collection tubes, and peripheral blood mononuclear cells (PBMCs) were isolated by standard Ficoll density gradient centrifugation. For NK cell activation and expansion, 6-well culture plates were pre-coated by adding 2 mL of Dulbecco’s phosphate-buffered saline (DPBS) and 100 μL of YC00A (NK activation reagent) to each well, followed by thorough mixing and incubation at 37 °C for 2 hours. After incubation, the supernatant was discarded, and wells were gently washed once with DPBS; the wash solution was then discarded. Subsequently, NK activation medium, 100 μL of YC00B (NK induction factor), 10% (v/v, 0.4 mL) autologous inactivated plasma, and 8 × 10^6^ isolated PBMCs were added to each pre-coated well to a final volume of 4 mL, corresponding to a seeding density of 2 × 10^6^ PBMCs/mL. From day 3 post-seeding, fresh activation medium was supplemented every other day, with mature activated NK cells harvested on day 14. To establish the miR-17-5p overexpressing NK cell model, on day 5 of *in vitro* activation culture, NK cells exhibiting good morphology and viability ≥ 90% were harvested and gently washed with pre-chilled DPBS. Cells were then resuspended in an appropriate volume of fresh NK activation medium supplemented with key cytokines, including IL-2 and IL-15. After counting, recombinant lentivirus carrying the human miR-17-5p sequence was added at an MOI of 20, along with Polybrene at the manufacturer-recommended concentration (final: 4–8 μg/mL), followed by gentle mixing. The cell suspension was incubated for 2 hours in a humidified incubator at 37 °C with 5% CO_2_. Subsequently, fresh activation medium was added to the recommended working volume for continuous culture. Lentiviral transduction efficiency was monitored by evaluating the GFP-positive rate under a fluorescence microscope every 72 hours.

### Isolation of CAR-T EVs

Seventy-two hours after lentiviral transduction of T cells, an equal volume of fresh KBM-581 medium supplemented with 200 IU/mL IL-2 was replenished every other day. After 14 days of CAR-T culture, culture supernatants were harvested, and CAR-T EVs were isolated by sequential differential centrifugation, with all steps conducted at 4 °C to preserve EV integrity: (1) centrifugation at 300 × *g* for 5 minutes to remove intact cells; (2) 2000 × *g* for 10 minutes to remove dead cells and large debris; (3) 10,000 × *g* for 30 minutes to remove large apoptotic vesicles, followed by discarding the precipitate; (4) filtration of the supernatant through a 0.45 μm low-protein-binding syringe filter to remove residual aggregates; (5) ultracentrifugation at 100,000 × *g* for 3 hours, then discarding the supernatant; (6) resuspension of the EV pellet in sterile EV-depleted PBS to obtain purified CAR-T EVs for downstream applications.

### Transmission electron microscopy morphological observation

Morphological identification of CAR-T EVs was performed by negative-stain TEM: (1) 10 μL of purified EV suspension was dropped onto a carbon-coated copper grid and allowed to settle for 1 min, then excess liquid was absorbed with filter paper; (2) 10 μL of uranyl acetate negative stain was dropped onto the grid and incubated for 1 min, then excess stain was removed with filter paper; (3) the grid was air-dried for several minutes at room temperature; (4) the morphology and size of CAR-T EVs were observed under a transmission electron microscope with an accelerating voltage of 80 kV.

### Nanoparticle tracking analysis

NTA was employed to assess the particle size distribution and concentration of CAR-T EVs. The procedure involved the following steps: (1) An appropriate aliquot of purified CAR-T EVs was diluted with particle-free PBS to achieve a suitable concentration for instrument detection; (2) Instrument performance was calibrated using standard reference particles prior to sample loading, and gradient dilutions were conducted to prevent clogging of the injection needle; (3) After detection, particle size and concentration data for CAR-T EVs were obtained and analyzed.

### Nano flow cytometry detection of CAR-T EV surface protein markers

Surface protein markers of CAR-T EVs were assessed using NanoFCM following this protocol: (1) 30 μL of purified CAR-T EVs was diluted to 90 μL with PBS, after which 30 μL of diluted EVs was mixed with 20 μL of fluorescence-conjugated antibodies targeting CD9, CD63, and CD81. The mixture was gently mixed and incubated in the dark at 37 °C for 30 minutes; (2) 1 mL of pre-chilled PBS was added, and the mixture was ultracentrifuged at 110,000 × *g* for 70 minutes at 4 °C using an ultracentrifuge rotor to remove unbound antibodies; (3) The supernatant was carefully discarded, and 1 mL of pre-chilled PBS was added, with the ultracentrifugation step repeated under the same conditions; (4) The supernatant was again carefully discarded, and the EV pellet was resuspended in 50 μL of pre-chilled 1 × PBS; (5) Instrument performance was verified with standard reference beads before sample loading, and all samples were serially diluted to avoid needle clogging; (6) Data on target protein marker expression were collected following sample detection.

### Western blotting

Total protein quantification of EV samples was conducted using the BCA colorimetric method according to the manufacturer’s instructions for the commercial BCA protein quantification kit. The EV samples were thoroughly mixed with an equal volume of detergent-containing lysis buffer and lysed in a 37 °C water bath for 30 minutes before quantification. A standard curve was created using bovine serum albumin (BSA) across a concentration gradient of 0 to 2 μg/μL, and the final absorbance reading was measured at 562 nm using a microplate reader.

Nanoflow cytometry enabled correlation calibration between particle number and protein concentration for all enrolled EV samples. The average particle-to-protein ratio of these EV samples was approximately (1.31 ± 0.33) × 10^10^ particles per microgram of total protein, with dosing concentrations in all functional experiments uniformly calibrated based on the total protein concentration.

Western blotting was performed to verify the expression of EV markers and target proteins, utilizing the following primary antibodies at their respective dilutions: anti-CD9, anti-CD63, anti-TSG101, anti-ALIX, anti-Calnexin, anti-NKG2D, anti-RUNX1, and GAPDH. Corresponding horseradish peroxidase (HRP)-conjugated secondary antibodies were employed for signal detection in accordance with standard protocols.

### Uptake detection of CAR-T EVs

NKG2D CAR-T EVs were labeled with PKH26 (1 μM, 30 min, 37 °C), purified via ultracentrifugation (110,000 × *g*, 2 h, 4 °C), and resuspended in PBS. Human HCC 97H cells were pre-labeled with PKH2 (2 μM, 30 min, RT) and seeded at a density of 2 × 10^4^ cells/well. Dose-dependent (0–100 μg/mL EV protein) and time-course (0–24 h) uptake assays were conducted, with non-internalized EVs removed by washing with ice-cold PBS. Cells were fixed in 4% PFA, counterstained with DAPI (1 μg/mL), and imaged using a Leica TCS SP8 confocal microscope. Internalized EVs were quantified using ImageJ, assessing mean fluorescence intensity per cell with a minimum of 200 cells per condition.

### Loading of miR-17-5p inhibitor into CAR-T EVs

CAR-T EVs (100 μg) were transfected with 20 pmol Cy3-labeled miR-17-5p inhibitor using the Exo-Fect™ Transfection Kit according to the manufacturer’s protocol. After incubation at 37 °C for 10 min, the reaction was terminated with ExoQuick-TC™, followed by precipitation of loaded EVs (13,000 × *g*, 3 min), washing, and resuspension in 300 μL PBS. RNA loading efficiency was confirmed through denaturing agarose gel electrophoresis (2% gel, SYBR Green I, 120 V, 40 min) using total RNA extracted from lysed EVs, with naked inhibitor and unloaded EVs as controls.

### Cytotoxicity assessment using the CCK-8 assay

The CCK-8 assay was used to detect cellular metabolic activity, which reflects the relative differences in cell viability between different treatment groups and yields relative proliferation activity readings. Human HCC cell lines or immortalized normal human liver epithelial THLE-2 cells were seeded at 8 × 10³ cells/well in 96-well plates and allowed to adhere overnight. The following day, the culture medium was replaced with fresh medium containing serially diluted CAR-T EVs (0, 10, 25, 50, 100, 200 μg/mL) based on protein quantification. Control wells received either medium only (blank) or medium without EVs (negative control). After a 24-h incubation at 37 °C with 5% CO_2_, the EV-containing medium was removed, cells were washed once with PBS, and 100 μL of fresh medium plus 10 μL of CCK-8 reagent (Dojindo, #CK04) were added to each well. Plates were incubated in the dark for 2 hours, and absorbance at 450 nm was measured using a BioTek Synergy H1 microplate reader.

### Real-time cell killing assay

NK cell–mediated cytotoxicity against HCC cells was evaluated in real time using the xCELLigence DP system (ACEA Biosciences, San Diego, CA, USA). Briefly, 97H or Huh7 cells were seeded at 8 × 10³ cells/well in E-Plate 16 PET plates (ACEA, #300600890) and allowed to adhere overnight in RPMI-1640 medium supplemented with 10% FBS at 37 °C with 5% CO_2_. Cell adhesion, spreading, and proliferation were monitored every 15 min via the integrated RTCA S16 Station until cells reached the log-phase growth plateau (typically 20–24 h post-seeding). Following this, the culture medium was carefully aspirated, and fresh medium containing allogeneic NK cells was added at an effector-to-target (E:T) ratio of 5:1. Control wells received medium only (target-cell control). The plate was returned to the station, and cell impedance (expressed as Cell Index) was recorded every 5 min for 72 h under standard culture conditions.

### IFN-γ ELISPOT assay

IFN-γ secretion by NK cells co-cultured with 97H or Huh7 cells was quantified using a human IFN-γ ELISPOT kit. Plates were pre-activated with RPMI-1640 (10 min, 37 °C), followed by washing. Target and allogeneic NK cells (isolated from healthy donor PBMCs; >90% CD56^+^CD3^–^) were seeded at an effector-to-target (E:T) ratio of 5:1 (5 × 10^4^ NK cells/well and 1 × 10^4^ target cells) in KBM-581 medium and incubated for 24 hours at 37 °C with 5% CO_2_. After washing, biotinylated anti–IFN-γ antibody (clone 7-B6-1, 1:100) and streptavidin–HRP (1:100) were sequentially applied for 1 hour each at 37 °C. Spots were developed using AEC substrate (10–20 min in the dark at 37 °C), imaged via a CTL ImmunoSpot S6 Analyzer, and quantified as spots per 10^5^ NK cells. Automatic enumeration employed a minimum diameter threshold of 50 µm. Controls included unstimulated NK cells (negative), PMA/ionomycin-stimulated NK cells (positive), and target-only wells (background). Experiments were conducted in triplicate wells and repeated independently three times.

### Zebrafish xenograft model

Wild-type AB zebrafish embryos at 48 hours post-fertilization (hpf) were microinjected with 200 Dil-labeled 97H cells into the yolk sac and incubated at 35 °C for 24 hours. At 72 hpf, Dio-labeled human NK cells (100 cells per embryo) were injected into the same site under three conditions: (1) untreated NK cells; (2) LV-miR–transduced NK cells; and (3) LV-miR NK cells pre-treated with 50 μg/mL EV-miR-17-5p inhibitor for 48 hours. Stereoscopic microscopy was performed at 2 and 24 hours post-NK cell injection. Tumor (Dil^+^ red) and NK cell (Dio^+^ green) areas within the yolk sac were quantified using ImageJ (v1.53t) with uniform threshold settings. Thirty embryos per group were analyzed, excluding malformed or leaky specimens.

### Statistical analysis

Data are presented as mean ± SD from at least three independent biological replicates. Normality (Shapiro–Wilk test) and homoscedasticity (Levene’s test) were confirmed prior to parametric analyses. Two-group comparisons employed two-tailed *t*-tests, while multi-group comparisons used one-way ANOVA with Tukey’s *post hoc* test. Statistical analyses were performed using GraphPad Prism 9.0.

## Results

### NKG2D-ligand–targeted third-generation CAR is efficiently expressed on primary human T cells

A third-generation CAR (4-1BB–CD3ζ) targeting stress-inducible NKG2D ligands (MICA/B, ULBP1–6) was designed, incorporating a human CD8α signal peptide, the extracellular domain of human NKG2D, a CD8α hinge, a CD28 transmembrane domain and intracellular domain (ICD), and intracellular 4-1BB and CD3ζ signaling domains (**Figure 1A**). An IRES–GFP cassette facilitated co-expression and tracking. Lentiviral transduction of primary T cells from healthy donors achieved > 95% GFP^+^ cells at 72 h post-transduction (**Figure 1B**), confirming efficient delivery and translation. Flow cytometry revealed robust surface expression of CAR-T cells at 57.17% ± 10.56% (**Figure 1C**). These data demonstrate high-efficiency, specific membrane expression of a functional, EV-compatible CAR-T platform.

**Figure 1:**
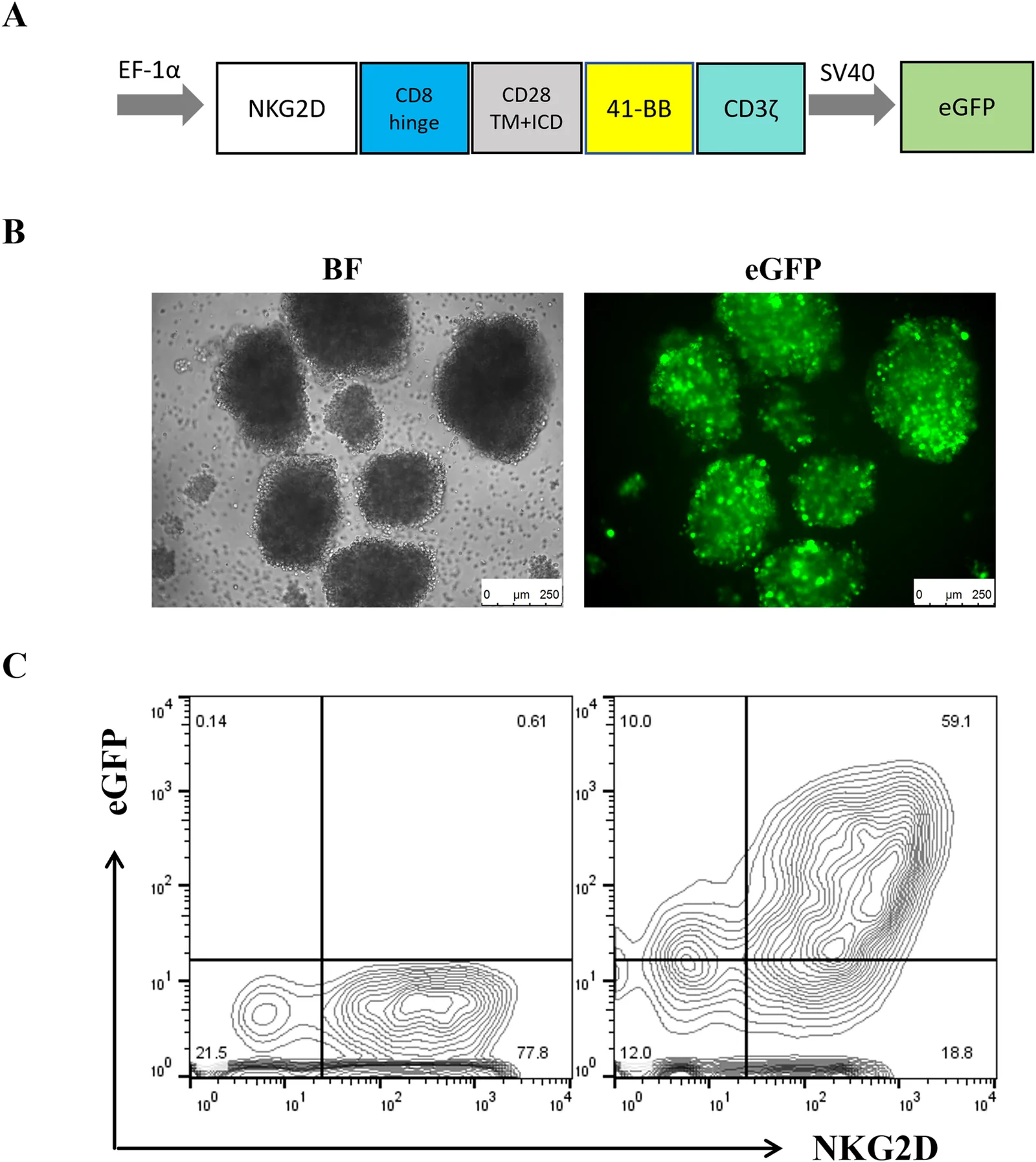
Validation of engineering and surface display for a third-generation NKG2D-ligand-targeted CAR in primary human T cells. **(A)** Diagrammatic outline of the third-generation lentiviral construct encoding the extracellular moiety of the human NKG2D-based CAR; **(B)** Representative fluorescence micrographs of CAR-transduced T cells captured 72 hours post-transduction: the left panel depicts the bright-field view, whereas the right panel exhibits the corresponding eGFP channel (scale bar = 250 µm); **(C)** Flow cytometry detected CAR-T transfection efficiency via APC anti-human NKG2D antibody and CAR-encoded GFP. The eGFP^+^/NKG2D^+^ double-positive rate from 3 independent donors was 57.17 ± 10.56%. Left: Isotype control. Right: upper-right quadrant shows the positive ratio of CAR-T cells.

### Isolation and characterization of CAR-T–derived extracellular vesicles

To isolate EVs from CAR-T cells, culture supernatants obtained from serum-free, 14-day cultures were processed through sequential differential ultracentrifugation (**Figure 2A**). TEM indicated that the purified vesicles displayed the characteristic cup-shaped morphology and intact lipid bilayer of small EVs (**Figure 2B**). NTA confirmed a homogeneous size distribution, with a peak between 50–150 nm and a mean particle diameter of 83.3 ± 2.1 nm (**Figure 2C**). Western blot analysis demonstrated strong enrichment of canonical EV protein markers (Alix, TSG101, CD9, Flotillin-1) and absence of the endoplasmic reticulum contaminant Calnexin, indicating high vesicle purity (**Figure 2D**). The engineered human NKG2D protein was robustly detected in these EVs, confirming successful packaging of the functional CAR extracellular domain. At the single-particle level, nano-flow cytometry (NanoFCM) was used to quantify surface tetraspanin expression on the isolated EV population, showing distinct positivity rates for CD9, CD63, and CD81 across the particle subset. (**Figure 2E**). Collectively, these orthogonal analyses—encompassing morphology, size, biochemical composition, and surface phenotype—establish that the isolated vesicles meet the defining criteria for small EVs as outlined in the MISEV2023 guidelines and contain the engineered targeting receptor, providing a validated platform for subsequent functional studies as targeted therapeutic vectors.

**Figure 2:**
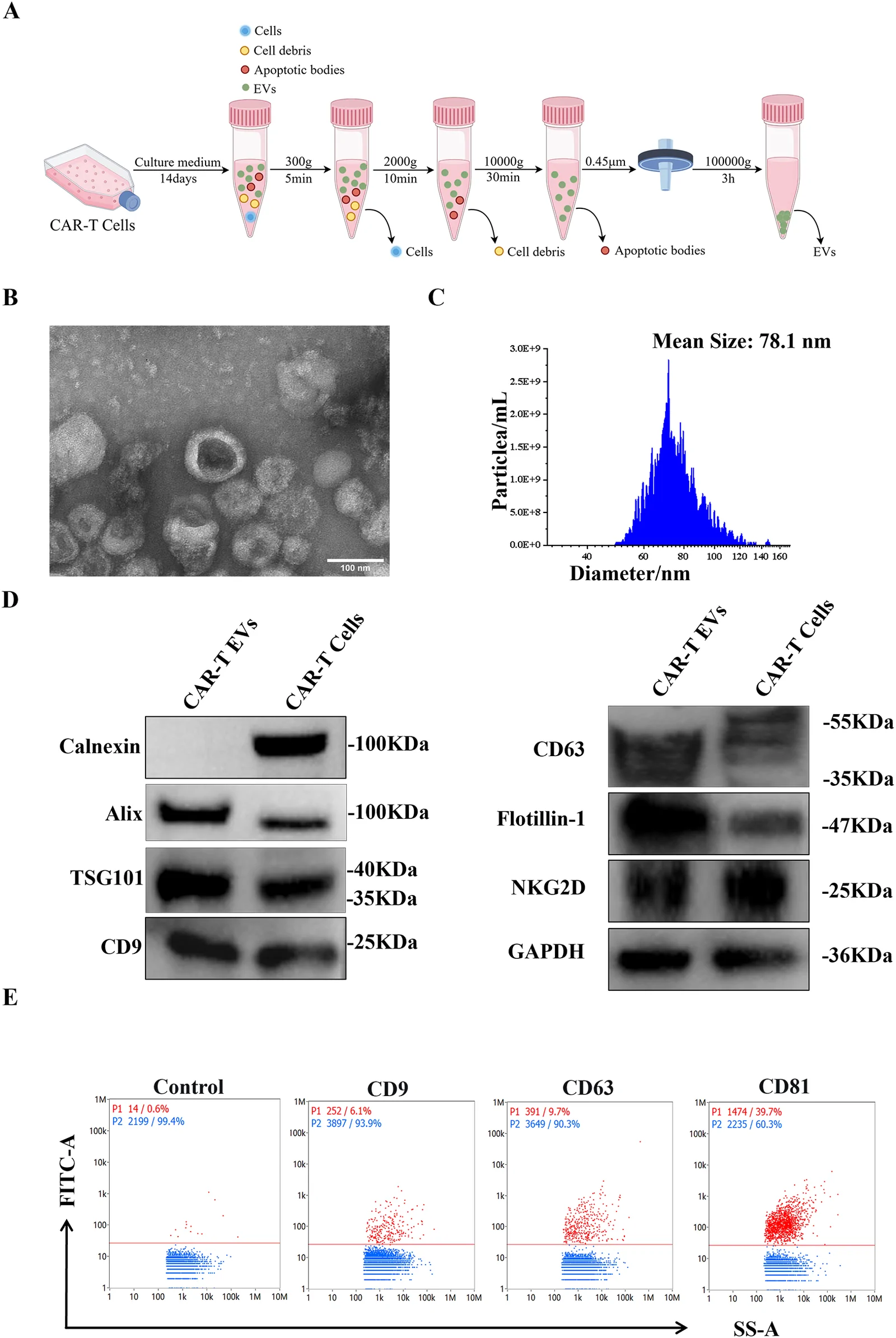
Characterization of extracellular vesicles derived from CAR-T cells. **(A)** A schematic diagram outlining the stepwise differential ultracentrifugation strategy utilized to isolate extracellular vesicles (EVs) from the supernatant of serum-free CAR-T cells; **(B)** A representative transmission electron microscopy (TEM) micrograph illustrating the characteristic cup-shaped morphology and preserved lipid bilayer structure of the purified EVs (scale bar = 100 nm); **(C)** Nanoparticle tracking analysis of EV size distribution indicated a modal size of 103 nm and a Z-average diameter of 83.3 ± 2.1 nm (mean ± SD, *n* = 3 independent preparations); **(D)** Western blot assessment of EV lysates targeting canonical EV markers alongside the engineered human NKG2D extracellular domain; **(E)** NanoFCM verification confirmed the surface presentation of the tetraspanins CD9, CD63, and CD81 on individual EV particles.

### Proteomic profiling of CAR-T EVs reveals an enriched functional repertoire with immunomodulatory potential

To comprehensively characterize the protein cargo and infer the functional basis of CAR-T EVs, label-free quantitative proteomics was performed, identifying a total of 3,456 proteins in the purified EV preparations. Comparative analysis with the proteome of parental CAR-T cells revealed that approximately 95% (3,286 proteins) of the EV proteome was shared, indicating substantial inheritance of parental cell components alongside a distinct EV packaging signature (**Figure 3A**).

**Figure 3:**
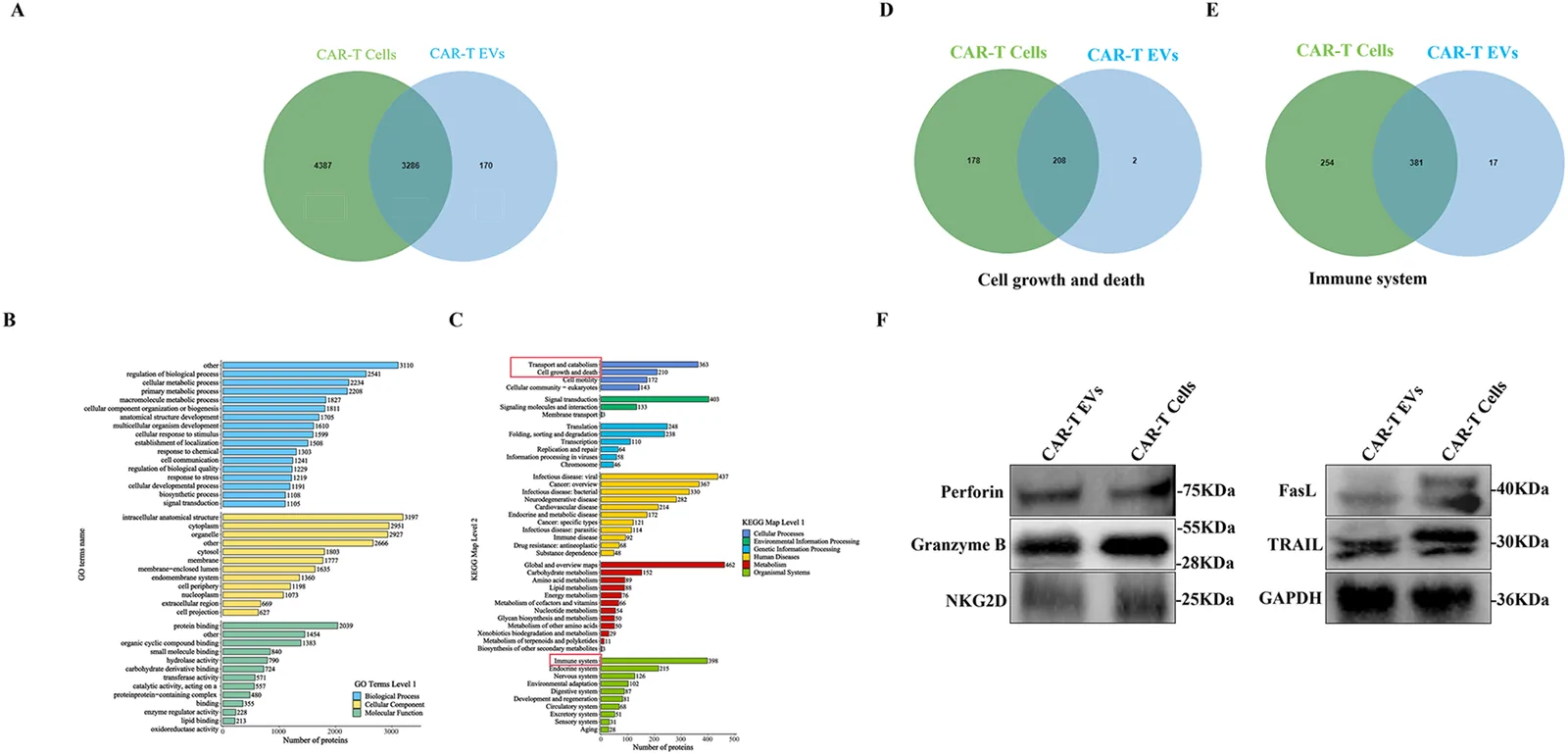
Proteomic landscape of CAR-T–secreted extracellular vesicles uncovers a functional arsenal dominated by immune-centric pathways. **(A)** Venn diagram juxtaposing the proteomic signatures of CAR-T EVs against their progenitor CAR-T cells; **(B)** Gene Ontology (GO) enrichment profiling of CAR-T EV-associated proteins. Bar graphs illustrate the most significantly enriched terms across Biological Process (BP), Cellular Component (CC), and Molecular Function (MF) domains; **(C)** Kyoto Encyclopedia of Genes and Genomes (KEGG) pathway enrichment mapping. The bar chart highlights significantly enriched pathway, annotating the count of contributing proteins per pathway, with the “Immune System” cluster distinctly emphasized; **(D)** Venn diagram contrasting proteomes of CAR-T EVs and parental CAR-T cells specifically within the KEGG “Cell growth and death” pathway; **(E)** Venn diagram comparing proteomic overlaps within the KEGG “Immune system” pathway; **(F)** Western blot assessment of EV lysates targeting cytotoxic effectors: perforin, granzyme B, Fas ligand, TNF-related apoptosis-inducing ligand, and the engineered NKG2D extracellular domain.

Gene Ontology (GO) enrichment analysis across biological processes, cellular components, and molecular functions provided a systematic view of the EV protein repertoire (**Figure 3B**). Proteins were significantly enriched in biological processes related to metabolism, biological regulation, cell communication, and response to stimuli. At the molecular function level, enriched terms included protein binding, organic compound binding, and catalytic activities (e.g., hydrolase and transferase activity). Cellular component analysis confirmed the expected subcellular origins, with strong enrichment for proteins localized to intracellular structures, the cytoplasm, and membrane systems.

KEGG pathway analysis further delineated the key biological processes associated with the EV proteome (**Figure 3C**). The most prominent enrichment was observed within the “Organismal Systems” category, where proteins involved in immune system pathways constituted the largest subset (398 proteins). Within “Cellular Processes,” notable enrichment occurred for proteins related to transport and catabolism (363 proteins), cell growth and death (210 proteins), and cell motility (172 proteins). Significant enrichment was also detected in pathways associated with signal transduction (“Environmental Information Processing”) and various metabolic pathways. Collectively, the proteomic data delineate a functionally diverse EV cargo with a pronounced representation of proteins implicated in immune regulation and intercellular communication.

To elucidate the origin of proteins underlying the key functional capacities of CAR-T EVs, their compositional relationship with parental CAR-T cells was analyzed within two critical functional modules identified by KEGG: “Cell growth and death” and “Immune system.” Venn diagram analysis revealed that of the 210 proteins enriched in the “Cell growth and death” pathway, 208 (99%) were shared with the parental cells (**Figure 3D**). Similarly, 381 out of the 398 proteins (96%) enriched in the “Immune system” pathway originated from parental CAR-T cells (**Figure 3E**). These data quantify a substantial parental contribution to the EV cargo within these functionally important pathways.

Based on the proteomic predictions, the presence of specific cytotoxic effector molecules in CAR-T EVs was experimentally validated by Western blot (**Figure 3F**). The analysis confirmed the expression of key cytotoxic proteins, including perforin, granzyme B, Fas ligand (FasL), and TNF-related apoptosis-inducing ligand (TRAIL). Consistent with earlier characterization, the extracellular domain of the engineered CAR (NKG2D) was also detected. These results provide direct protein-level evidence that CAR-T EVs package and carry a repertoire of critical immune recognition and effector molecules derived from their parental cells.

### Internalization and bioactivity of NKG2D CAR-T EVs in hepatocellular carcinoma cells

To characterize the cellular uptake kinetics of CAR-T EVs, they were labeled with the lipophilic dye PKH26 (red fluorescence) and co-cultured with PKH2-labeled (green cytoplasm) and DAPI-stained (blue nucleus) 97H cells. A concentration gradient assay (0, 10, 25, 50, and 100 µg/mL EVs for 24 h) revealed a dose-dependent increase in intracellular red fluorescence intensity, which plateaued at 50 and 100 µg/mL, indicating saturation of the uptake process (**Figure 4A**). A time-course assay with 50 µg/mL EVs at 0, 6, 12, and 24 h demonstrated significant time-dependent accumulation of EV-associated fluorescence within 97H cells (**Figure 4B**). Based on these data, 50 µg/mL for 24 h was selected as the standard condition for subsequent functional assays. Confocal microscopy confirmed efficient internalization, showing abundant punctate red fluorescence localized within the cytoplasm of 97H cells (**Figure 4C**).

**Figure 4:**
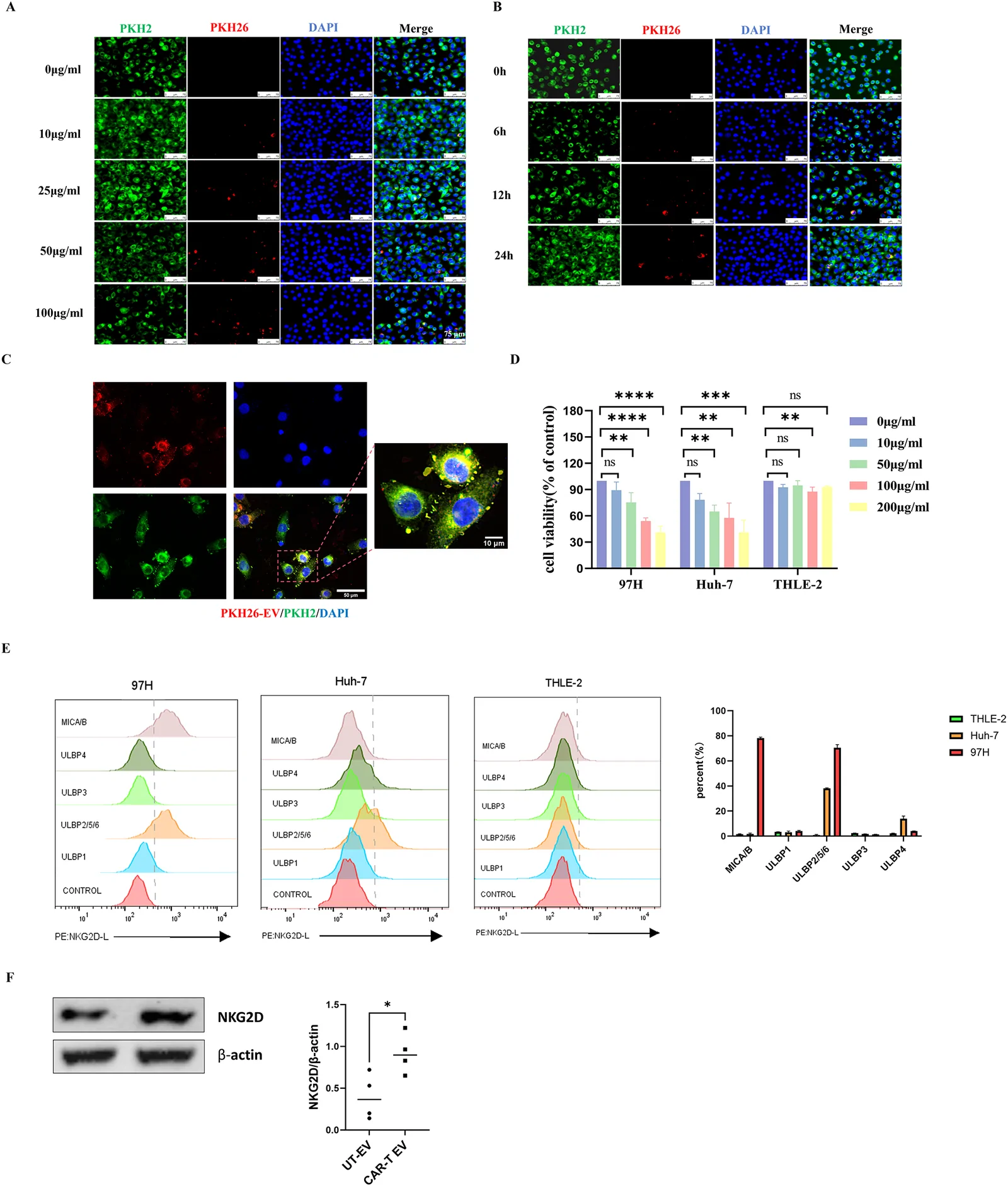
NKG2D-orchestrated targeted internalization and discriminatory cytotoxicity of CAR-T–Derived extracellular vesicles against hepatocellular carcinoma cells. **(A)** Dose-responsive internalization analysis. 97H cells were exposed to PKH26-tagged CAR-T EVs for 24 h. Fluorescence microscopy revealed a graded increase in intracellular PKH26 intensity. **(B)** Temporal uptake profiling. 97H cells were incubated with 50 µg/mL PKH26-labeled EVs. **(C)** Confocal microscopy confirmation of vesicle entry; **(D)** Normal human liver epithelial cells (THLE-2) and hepatocellular carcinoma cell lines (97H and Huh7) were treated with increasing doses of CAR-T EVs and subsequently evaluated using the CCK-8 assay. **(E)** Flow cytometry analysis of NKG2D ligand expression in MHCC-97H, Huh7, and THLE-2 cells. Both MHCC-97H and Huh7 cells expressed a subset of NKG2D ligands, whereas THLE-2 cells exhibited no detectable significant NKG2D ligand expression. **(F)** Parallel Western blot analysis of extracellular vesicles derived from un-transduced activated T cells (UT-EV) and CAR-T cells. All samples from the two groups were processed and detected synchronously under strictly identical experimental conditions. The NKG2D signal in the UT-EV group remained at the low baseline level of endogenous expression, whereas the NKG2D signal intensity in CAR-T EVs showed clear and statistically significant specific enrichment. Results are depicted as mean ± SD derived from five independent biological replicates. Statistical significance was determined using one-way ANOVA followed by Tukey’s *post hoc* test. ns, not significant; *p < 0.05; **p < 0.01; ***p < 0.001; ****p < 0.0001.

The functional impact of CAR-T EVs on cell viability was assessed using the CCK-8 assay, a method that measures metabolic activity as a proxy for relative cell viability. Primary normal human liver epithelial cells (THLE-2) and two HCC cell lines (97H and Huh7) were treated with increasing concentrations of EVs (0–200 µg/mL) for 48 h. No significant reduction in THLE-2 metabolic activity (relative viability) was observed across all tested concentrations. In contrast, both 97H and Huh7 cells exhibited dose-dependent reduction in relative metabolic readout, with significant reductions in CCK-8 signals beginning at 25 µg/mL and reaching >50% inhibition at 100 µg/mL (**Figure 4D**). Flow cytometry validated key NKG2D ligand expression in MHCC-97H, Huh7, and the human normal liver cell line THLE-2, which served as a negative control. Both hepatoma cell lines, MHCC-97H and Huh7, expressed NKG2D ligands, with only a small proportion of subtypes showing no detectable expression. In contrast, THLE-2 cells exhibited no significant expression of any NKG2D ligands, consistent with their normal liver cell identity (**Figure 4E**). To explicitly distinguish the exogenous NKG2D domain on the CAR molecule from the endogenously expressed NKG2D protein in T cells, and to verify the specific integration of CAR molecules into EVs, EVs derived from untransduced activated T cells were introduced as a parallel negative control. Results showed that the NKG2D signal in the UT-EV group remained at low baseline levels of endogenous expression, while the NKG2D signal in CAR-T EVs exhibited significant specific enrichment. This finding confirms that the dominant NKG2D signal detected in CAR-T EVs originates from the overexpressed CAR construct, rather than the background endogenous NKG2D protein carried by EVs released from activated T cells. These results indicate that exposure to CAR-T EVs leads to distinct response profiles in NKG2D ligand-positive HCC cells, whereas normal hepatocytes demonstrate minimal corresponding phenotypic changes after the same treatment.

### Engineered encapsulation and intracellular delivery of miR-17-5p inhibitor by CAR-T EVs

To quantify the encapsulation efficiency of *miR-17-5p inhibitor-Cy3* into CAR-T EVs, a fluorescence intensity–concentration standard curve was first established. Serial dilutions of the inhibitor (0–1,000 nM) were measured by fluorometry, yielding a linear regression equation of *y = 43.54x + 100.8* (*R*^2^ = 0.9989), confirming high assay linearity and reproducibility (**Figure 5A**).

**Figure 5:**
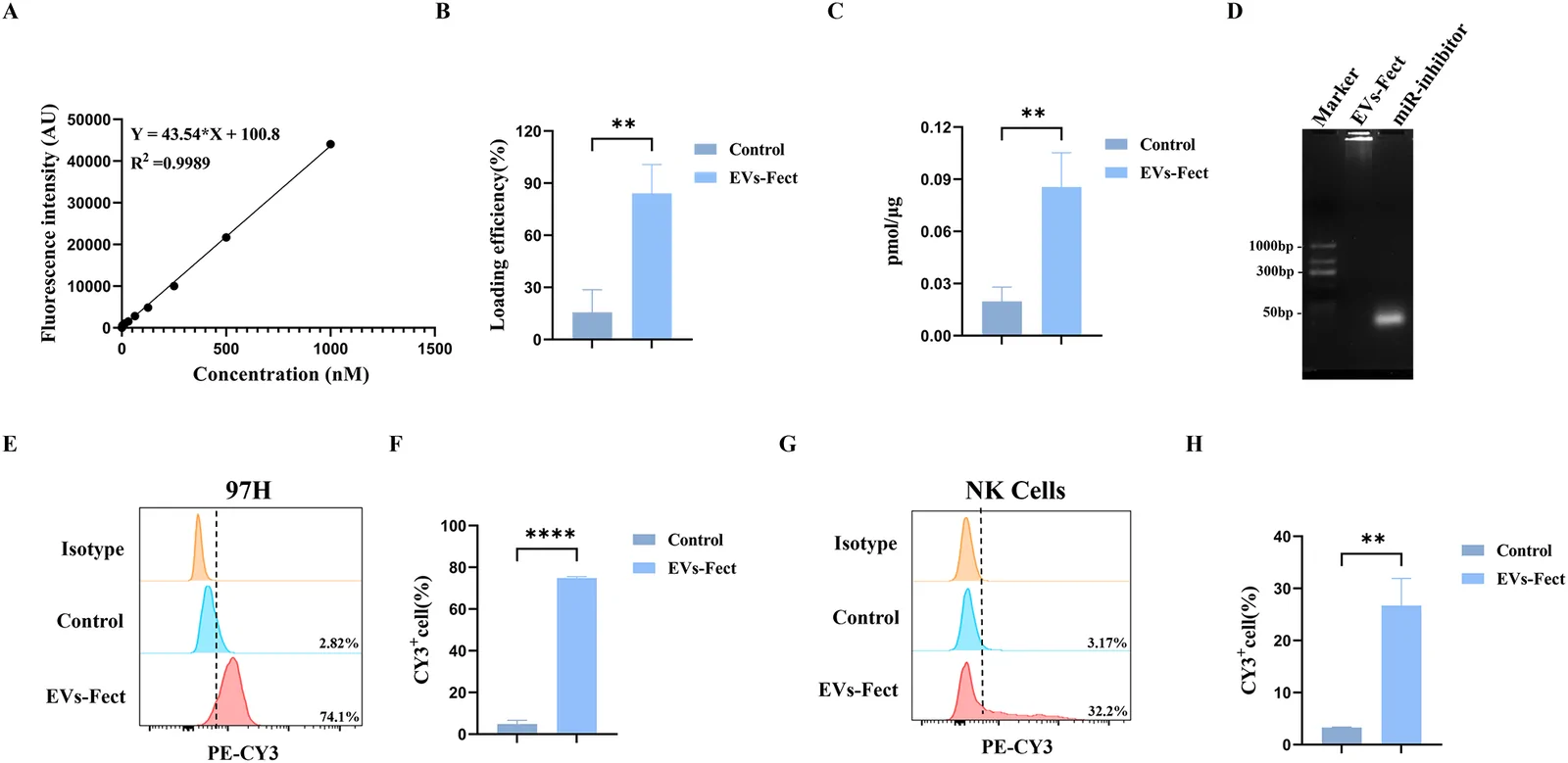
Strategic encapsulation and potent intracellular transport of miR-17-5p inhibitor-Cy3 via CAR-T–derived extracellular vesicles. **(A)** Calibration curve for quantitative analysis. **(B)** Encapsulation efficacy. The proportion of miR-17-5p inhibitor-Cy3 sequestered within EVs was assessed by fluorescence spectroscopy: Control cohort (direct co-incubation in PBS) versus EVs-Fect cohort (Exo-Fect™-facilitated loading). **(C)** Payload capacity. The quantity of miR-17-5p inhibitor-Cy3 loaded per µg of EV protein (pmol/µg) was derived from the calibration curve. **(D)** Agarose gel electrophoresis verifying intraluminal confinement. Unbound miR-17-5p inhibitor-Cy3 migrated toward the anode, whereas inhibitor-laden EVs retained >95% of the Cy3 signal within the wells. **(E, F)** Targeted delivery to 97H hepatocellular carcinoma cells. Cells were exposed to EVs from the Control or EVs-Fect cohorts for 24 h. **(E)** Representative flow cytometry dot plots illustrating Cy3^+^ cells (gated on viable, single cells). **(F)** Quantitative assessment of Cy3^+^ 97H cells (%). **(G, H)** Delivery to primary human natural killer (NK) cells. NK cells were incubated with EVs from the EVs-Fect cohort for 24 h. **(G)** Representative flow cytometry dot plots. **(H)** Quantification of Cy3^+^ NK cells (%). *n* = 4 independent experiments. Statistical significance was established using an unpaired two-tailed Student’s *t*-test **(B, C, F, H)**. Data are expressed as mean ± SD. ns, *p* > 0.05, **p < 0.01, ****p < 0.0001.

EVs were engineered using Exo-Fect™ reagent according to the manufacturer’s protocol, including two control groups: (i) *Direct co-incubation* (EVs + inhibitor in PBS, no reagent) and (ii) *Exo-Fect–mediated loading* (EVs + inhibitor + Exo-Fect™). Fluorescence quantification revealed that Exo-Fect–mediated loading achieved an encapsulation efficiency of 84.17% ± 16.61%, significantly higher than the 15.63% ± 12.90% observed in the direct co-incubation group (*p* < 0.01; **Figure 5B**). The average loaded dose was 0.086 pmol inhibitor per µg EV protein in the Exo-Fect group, representing a 4.3-fold increase compared to the control group (**Figure 5C**).

To differentiate between true intraluminal encapsulation and surface adsorption, agarose gel electrophoresis was performed. Free *miR-17-5p inhibitor-Cy3* migrated robustly toward the anode, whereas inhibitor-loaded EVs retained >95% of the Cy3 signal within the loading wells (**Figure 5D**), confirming successful intraluminal encapsulation rather than passive membrane association.

With efficient engineering established, functional intracellular delivery was assessed. 97H cells were incubated with engineered EVs for 24 h, and Cy3-positive cells were quantified by flow cytometry. The Exo-Fect–engineered EVs induced a robust intracellular delivery efficiency of 74.9% ± 0.64%, compared to only 4.86% ± 1.77% in the direct co-incubation group (*p* < 0.001; **Figures 5E, F**). To evaluate cellular tropism beyond tumor cells, the same engineered EVs were co-cultured with primary human NK cells. Flow cytometric analysis indicated that 26.7% ± 5.23% of NK cells were Cy3-positive after 24 h (**Figures 5G, H**). These data demonstrate that Exo-Fect–engineered CAR-T EVs achieve high-efficiency, intraluminal encapsulation of miR-17-5p inhibitor, mediating potent delivery to both malignant hepatocytes and primary immune effector cells.

### Structural and molecular integrity of CAR-T EVs Is preserved following Exo-Fect™-mediated miR-17-5p inhibitor loading

To assess whether Exo-Fect™-mediated loading compromises the structural or molecular integrity of CAR-T EVs, comparisons were made between unloaded EVs (Control group) and miR-17-5p inhibitor-Cy3-loaded EVs (EVs-Fect group) across three orthogonal parameters: morphology, size distribution, and canonical EV marker expression. TEM revealed that both groups exhibited the characteristic cup-shaped, bilayer membrane morphology typical of small EVs (**Figure 6A**). NTA was performed at a standardized concentration of 1 × 10^9^ particles/mL in PBS. The mean modal diameter was 83.3 ± 2.1 nm for Control EVs and 84.4 ± 1.9 nm for EVs-Fect EVs (**Figure 6B**), indicating no significant shift in size distribution upon loading. Western blot analysis confirmed robust expression of canonical EV markers CD63, CD9, and TSG101 in both groups (**Figure 6C**). These data demonstrate that Exo-Fect™-mediated loading preserves the structural morphology, hydrodynamic size profile, and core molecular signature of CAR-T EVs.

**Figure 6:**
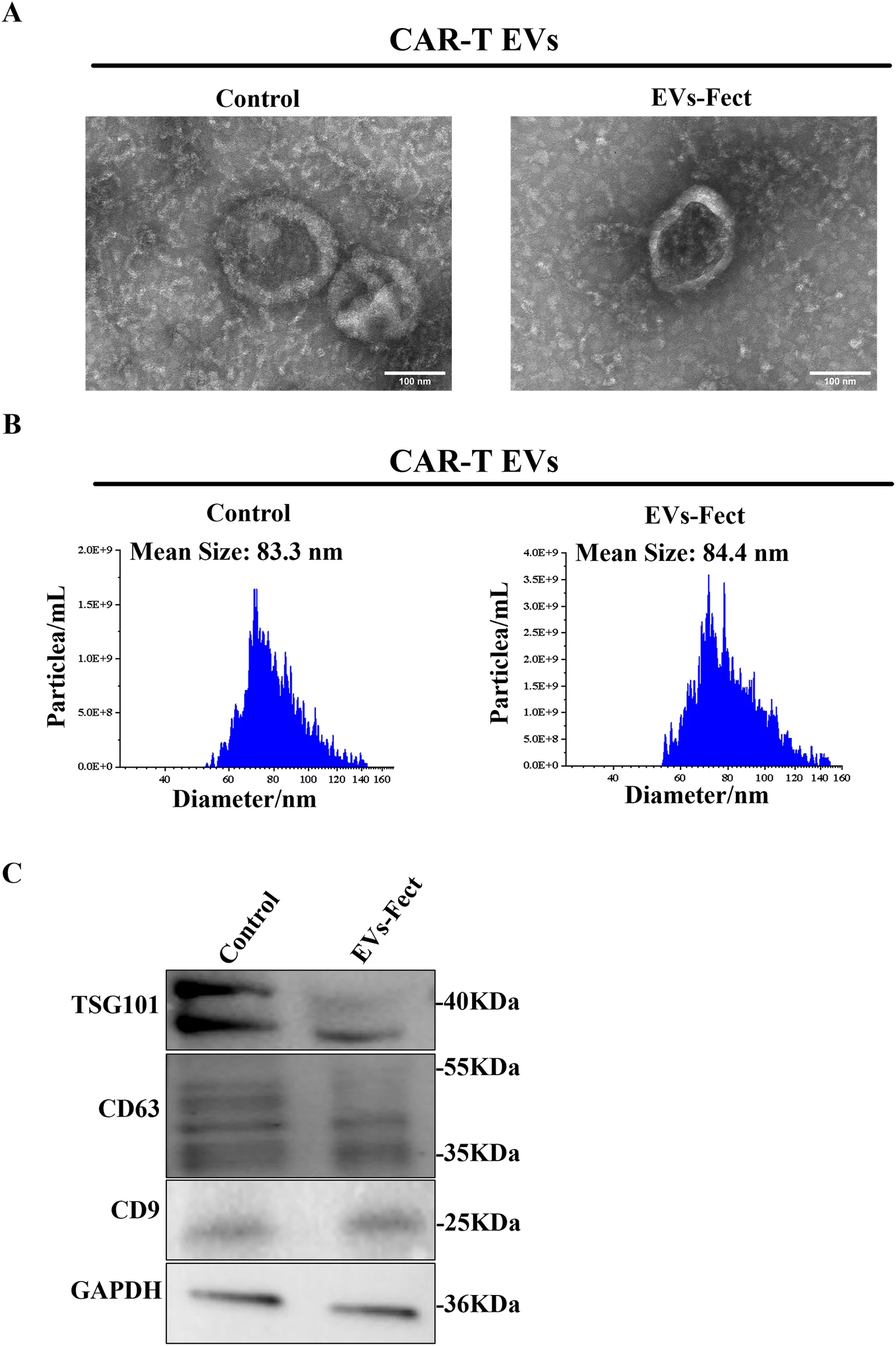
Structural integrity and core molecular signature of CAR-T EVs are unaltered by Exo-Fect™-mediated miR-17-5p inhibitor loading. **(A)** Transmission electron microscopy (TEM) micrographs reveal unloaded CAR-T EVs (Control group) alongside EVs loaded with miR-17-5p inhibitor-Cy3 (EVs-Fect group). Scale bar = 100 nm. Both populations exhibit the characteristic cup-shaped, bilayered architecture typical of small EVs. **(B)** Nanoparticle tracking analysis (NTA) delineates size distribution profiles. **(C)** Western blot interrogation confirms the presence of canonical EV markers CD63, CD9, and TSG101, with GAPDH employed as a loading reference.

### CAR-T EVs loaded with miR-17-5p inhibitor reinvigorate NK cell function via targeted restoration of the RUNX1/NKG2D axis

To establish a model of dysfunctional NK cells relevant to the HCC microenvironment, primary human NK cells were transduced with a lentiviral vector encoding miR-17-5p and GFP. At 72 hours post-transduction, over 50% of cells exhibited robust GFP fluorescence (**Figures 7A, B**), confirming efficient transduction. The proportion of CD3^–^CD56^+^ NK cells remained unchanged between transduced and untransduced controls (**Figure 7C**), indicating preservation of the core immunophenotype, and this population was designated LV-miR NK cells.

**Figure 7:**
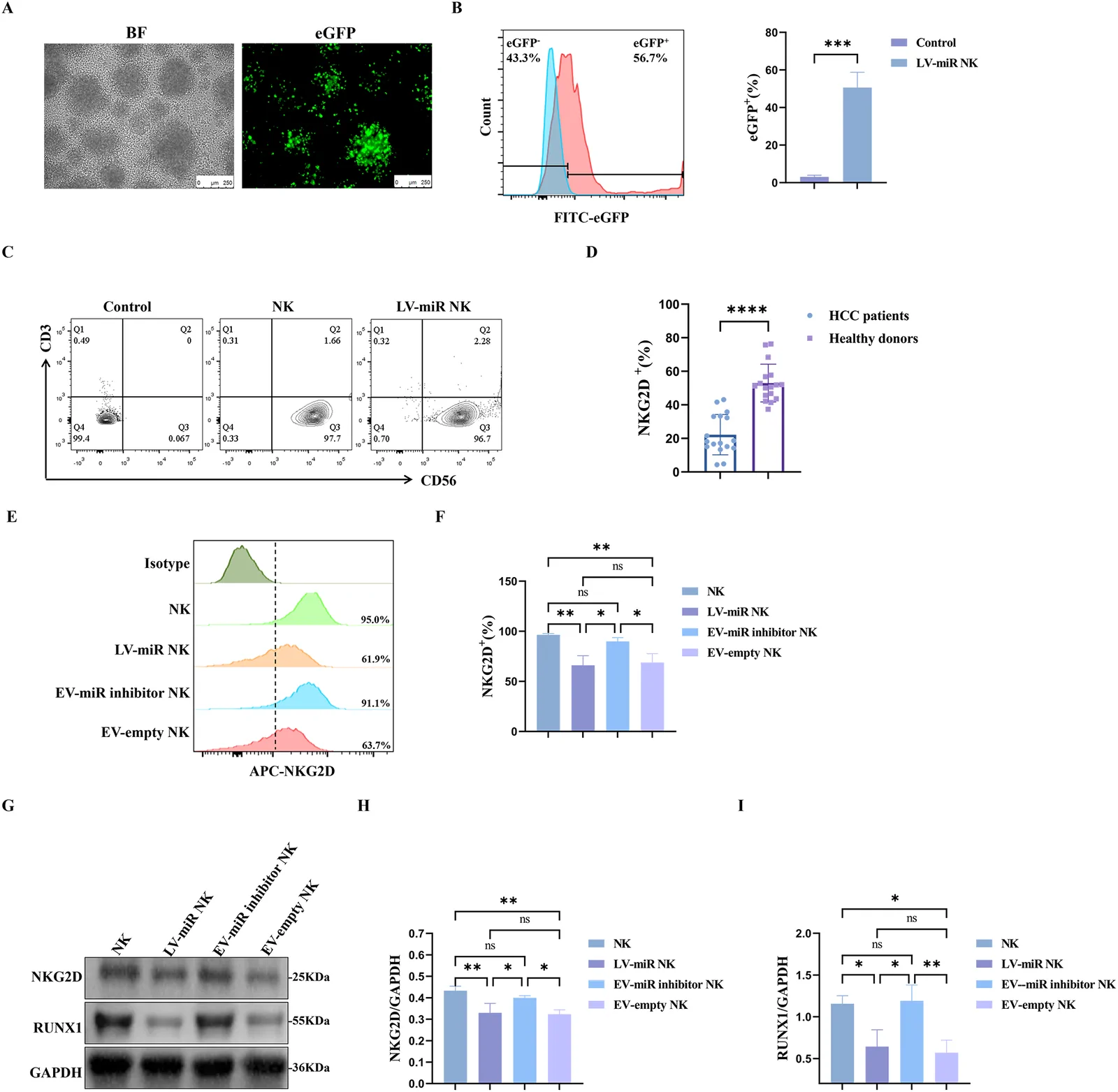
CAR-T EVs loaded with miR-17-5p inhibitor reinvigorate NK cell function via RUNX1/NKG2D axis restoration. **(A)** Fluorescence microscopy images of primary human NK cells transduced with miR-17-5p-GFP lentivirus for 72 h. Left: bright-field image; right: GFP fluorescence image. Scale bar = 250 µm. **(B)** Flow cytometry quantification of GFP^+^ cells among CD3^–^CD56^+^ NK cells at 72 h post-transduction. **(C)** Flow cytometry analysis of CD3^–^CD56^+^ NK cell frequency in untransduced (NK control) and miR-17-5p-transduced (LV-miR NK) cultures. No significant difference was observed (*p* = 0.72, unpaired two-tailed Student’s *t*-test). **(D)** Flow cytometry assessment of NKG2D surface expression on CD3^–^CD56^+^ NK cells from 17 healthy donors and 17 patients with HCC; **(E, F)** Functional reinvigoration assay. LV-miR NK cells were co-cultured for 48 h. Untreated NK cells served as baseline control (NK control). **(E)** Representative flow cytometry dot plots (gated on CD3^–^CD56^+^ viable single cells). **(F)** Quantification of NKG2D^+^ percentage. **(G–I)** Western blot analysis of NKG2D and RUNX1 protein levels. Equal amounts of total protein (20 µg/lane) were loaded. **(G)** Representative blots. **(H)** Quantification of NKG2D protein normalized to GAPDH. **(I)** Quantification of RUNX1 protein normalized to GAPDH. Data are presented as mean ± SD from *n* = 3 independent experiments. Statistical significance was determined by one-way ANOVA with Tukey’s multiple comparisons test as in **(F, H, I)**. ns, *p* > 0.05; *p < 0.05; **p < 0.01; ***p < 0.001; ****p < 0.0001.

Flow cytometry analysis of peripheral blood from 17 patients with HCC revealed significantly reduced NKG2D expression on CD3^–^CD56^+^ NK cells compared to healthy donors (**Figure 7D**). To test whether miR-17-5p inhibition could reverse this defect, LV-miR NK cells were subjected to four conditions: (i) untreated NK cells (NK control); (ii) LV-miR NK cells (dysfunctional model); (iii) LV-miR NK cells co-cultured with miR-17-5p inhibitor-Cy3-loaded CAR-T EVs (EV-miR inhibitor); and (iv) LV-miR NK cells co-cultured with unloaded EVs (EVs-empty). All co-cultures were performed for 48 hours. Flow cytometry showed that NKG2D surface expression was significantly suppressed in LV-miR NK cells compared to NK control (*p* < 0.01). Treatment with EV-miR inhibitor restored NKG2D levels to near baseline, while EVs-empty treatment induced no significant change (**Figures 7E, F**). Western blot analysis confirmed parallel changes in protein abundance: LV-miR NK cells exhibited markedly reduced NKG2D and RUNX1 protein levels, while EV-miR inhibitor treatment significantly increased both proteins (**Figures 7G–I**). These data demonstrate that miR-17-5p inhibitor-loaded CAR-T EVs specifically reinvigorate NK cell function by restoring the RUNX1-dependent NKG2D expression axis.

### CAR-T EVs loaded with miR-17-5p inhibitor rescue and potentiate NK cell function against HCC

Real-time cytotoxicity against HCC cells was assessed using the xCELLigence RTCA DP system. 97H or Huh7 cells were seeded in E-plates and allowed to adhere for 16 hours. Effector cells were added according to different experimental groups, which were set up as follows: Control group (tumor cells alone), untreated NK group, corresponding vehicle-treated NK group, empty EVs-preconditioned NK group, free miR-17-5p inhibitor-treated NK group, LV-miR-preconditioned NK group, and EV-miR inhibitor-preconditioned NK group. The effector-to-target (E:T) ratio was uniformly set at 5:1 for all groups.

The Control group showed progressive CI increase, confirming proliferation competence. The untreated NK, corresponding vehicle NK, empty EVs NK, and free miR-17-5p inhibitor NK groups exhibited higher Cell Index (CI) values than the Control group, without statistically significant differences among them (**Supplementary Figure 1**). These results exclude potential interfering effects from the EV vehicle and the free miR-17-5p inhibitor itself.

Compared to untreated NK cells, LV-miR NK cells exhibited significantly reduced cytolytic activity against both 97H and Huh7 cells, as indicated by higher CI values at all time points (*p* < 0.001). Treatment with EV-miR inhibitor fully rescued this defect; the CI kinetics of EV-miR inhibitor NK cells overlapped with those of NK control, indicating complete functional recovery (**Figures 8A, B**).

**Figure 8:**
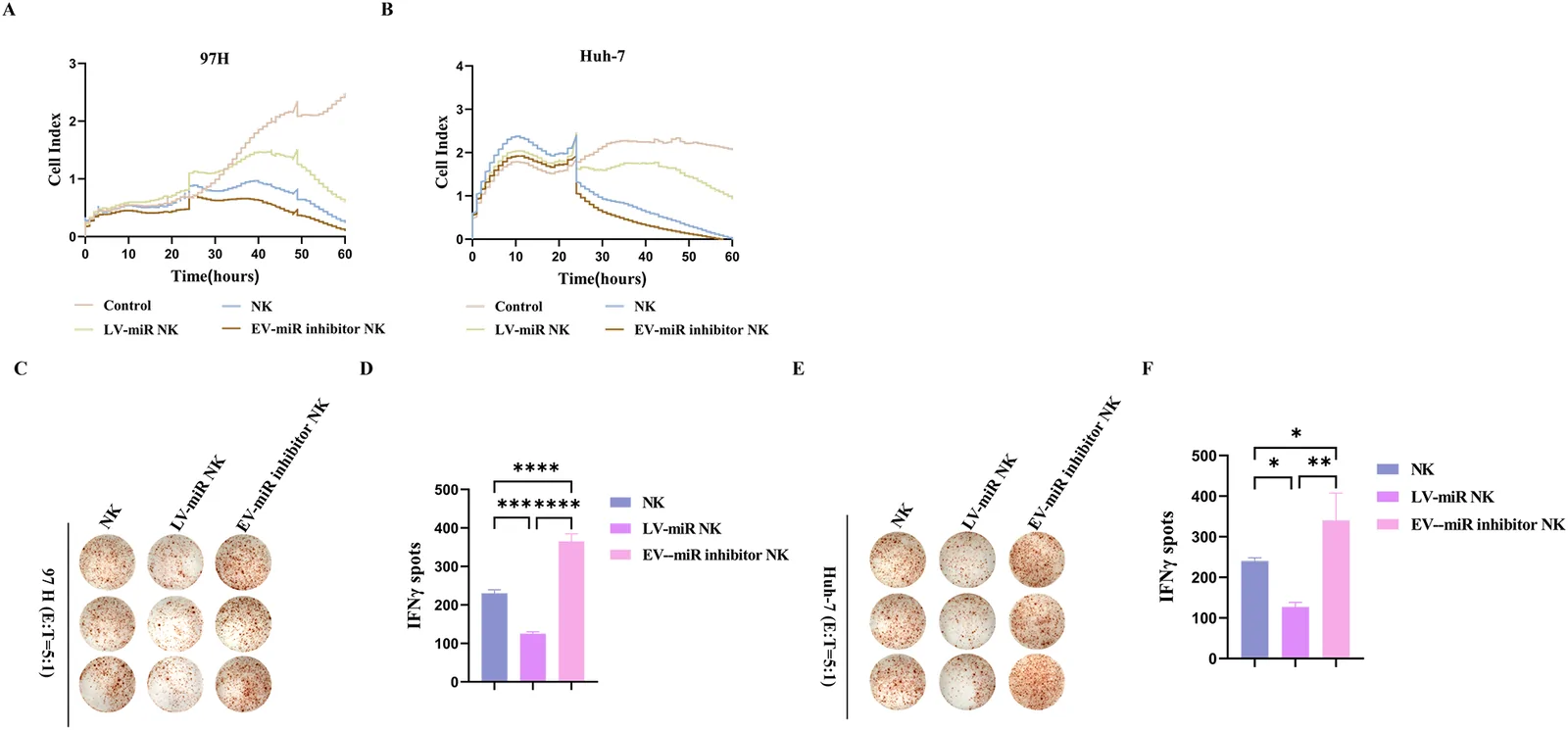
CAR-T EVs loaded with miR-17-5p inhibitor rescue and potentiate the antitumor function of NK cells. **(A, B)** Real-time assessment of NK cell cytotoxicity against hepatocellular carcinoma (HCC) cell lines using the xCELLigence RTCA DP system. **(C–F)** IFN-γ secretion profile of NK cells in response to HCC cells, assessed by ELISPOT. **(C, E)** Representative ELISPOT well images. **(D, F)** Quantification of spot-forming cells (SFCs) per 1 × 10^5^ NK cells. Data are presented as mean ± SD from *n* = 3 independent experiments. Statistical significance was determined by one-way ANOVA with Tukey’s *post hoc* test for multiple comparisons. ns, not significant; *p < 0.05; **p < 0.01; ***p < 0.001; ****p < 0.0001.

To evaluate cytokine-mediated effector function, IFN-γ secretion was quantified by ELISPOT following the co-culture of NK cells with 97H or Huh7 cells (E:T = 5:1). LV-miR NK cells produced significantly fewer IFN-γ spots than NK control (*p* < 0.01). Strikingly, EV-miR inhibitor NK cells secreted significantly more IFN-γ than NK control against both tumor lines (*p* < 0.001; **Figures 8C–F**). These data demonstrate that miR-17-5p inhibitor-loaded CAR-T EVs not only rescue but also potentiate the cytolytic and cytokine effector functions of NK cells in HCC.

### CAR-T EVs loaded with miR-17-5p inhibitor mediate *in vivo* functional reconstitution of NK cells in a zebrafish HCC xenograft model

To assess the *in vivo* impact of *miR-17-5p inhibitor*-loaded CAR-T EVs on NK cell function, a xenograft model of HCC was established in AB-strain zebrafish embryos at 48 hpf. DiI-labeled 97H cells were microinjected into the yolk sac. At 24 hours post-tumor injection, DiO-labeled NK cells from different experimental groups were co-injected into the same anatomical site. Live fluorescence imaging was performed using a stereoscopic microscope at 2 hours (baseline) and 24 hours (endpoint) post-NK injection. Tumor burden (DiI^+^ area, µm^2^) and NK cell persistence (DiO^+^ area, µm^2^) were quantified in maximum-intensity projections using ImageJ (v1.54f) with consistent intensity thresholds across all groups (*n* = 15–18 embryos per group; three independent experiments) (**Figures 9A, B**). Under strictly unified imaging parameters, changes in the coverage area of DiO-labeled NK cells or DiI-labeled 97H cells in zebrafish indicated corresponding increases or decreases in local cell accumulation levels within this model.

**Figure 9:**
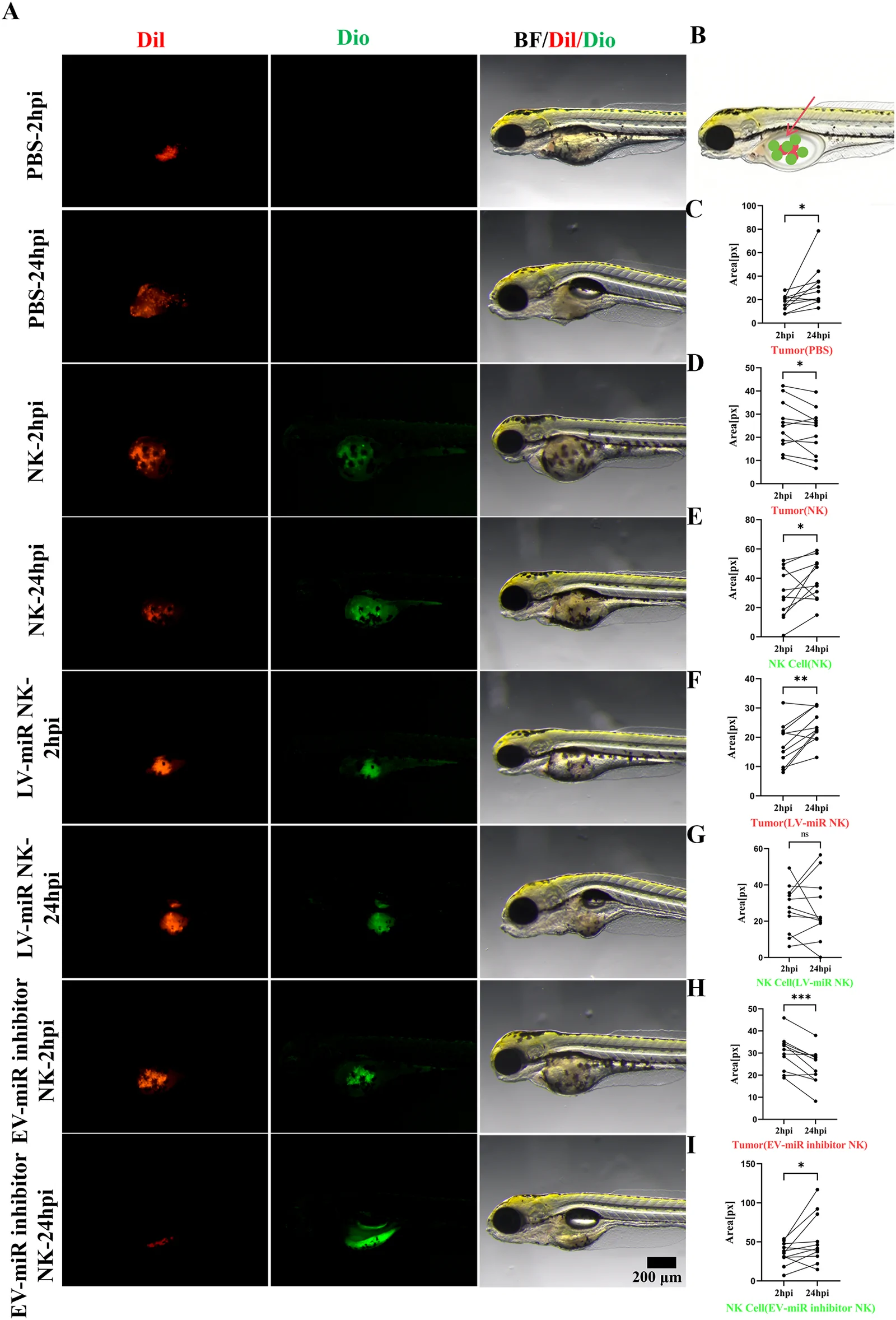
CAR-T EVs loaded with miR-17-5p inhibitor restore *in vivo* tumor burden control and NK cell persistence in a zebrafish HCC xenograft model. **(A)** Schematic of the zebrafish xenograft model. DiI-labeled (red) 97H cells were microinjected into the yolk sac of AB-strain zebrafish embryos at 48 hpf. At 24 h post-tumor injection, DiO-labeled (green) NK cells were co-injected into the same anatomical site. Live fluorescence imaging was performed at 2 h (baseline) and 24 h (endpoint) post-NK injection using a stereoscopic microscope. Scale bar = 100 µm. **(B)** Anatomical diagram indicating the yolk sac injection site. **(C–I)** Quantification of tumor burden (DiI^+^ area, µm^2^) and NK cell persistence (DiO^+^ area, µm^2^) in maximum-intensity projections. **(C)** PBS control group (tumor cells only). **(D, E)** NK control group: DiI^+^ area **(D)** and DiO^+^ area **(E)** at 2 h versus 24 h. **(F, G)** LV-miR NK group: DiI^+^ area **(F)** and DiO^+^ area **(G)** at 2 h versus 24 h. **(H, I)** EV-miR inhibitor NK group: DiI^+^ area **(H)** and DiO^+^ area **(I)** at 2 h versus 24 h. Data are presented as mean ± SD. For longitudinally collected fluorescence area data from the same individual embryo at multiple time points, statistical differences were evaluated using a two-tailed paired Student’s *t*-test. ns, not significant; *p < 0.05; **p < 0.01; ***p < 0.001.

At 24 hours post-NK injection, PBS control embryos exhibited a significant increase in DiI^+^ area compared to baseline (2 hours), confirming progressive tumor growth (*p* < 0.05; **Figure 9C**). NK control embryos showed a significant decrease in DiI^+^ area (*p* < 0.05; **Figure 9D**) and a significant increase in DiO^+^ area (*p* < 0.05; **Figure 9E**), indicating baseline antitumor activity and *in vivo* NK cell expansion. In contrast, LV-miR NK embryos displayed a significant increase in DiI^+^ area (*p* < 0.01; **Figure 9F**) and no increase in DiO^+^ area (*p >*0.05; **Figure 9G**), demonstrating functional impairment. Critically, embryos receiving EV-miR inhibitor NK cells exhibited a significant reduction in DiI^+^ area (*p* < 0.001; **Figure 9H**) and a corresponding increase in DiO^+^ area (*p* < 0.05; **Figure 9I**), demonstrating that *miR-17-5p inhibitor*-loaded CAR-T EVs fully restore tumor burden control and NK cell persistence in this *in vivo* HCC model.

## Discussion

CAR-T cell therapy has demonstrated remarkable clinical efficacy in hematologic malignancies, achieving complete remission rates exceeding 90% in patients with B-cell acute lymphoblastic leukemia (24, 25). However, its application in solid tumors is constrained by significant challenges, including the dense stromal barrier and the immunosuppressive TME, which drive rapid CAR-T cell dysfunction and exhaustion (2, 26). Consequently, novel therapeutic strategies capable of overcoming these limitations are critically needed. In this context, NKG2D ligand-targeted third-generation CAR-T cells were successfully generated, along with the isolation of corresponding CAR-T EVs. These vesicles exhibited typical EV characteristics, carried key cytotoxic effectors, and demonstrated significant killing effects on HCC cells while causing no harm to normal hepatocytes. Moreover, miR-17-5p inhibitor-loaded CAR-T EVs restored the function of dysfunctional NK cells and ultimately suppressed tumor growth in HCC preclinical models.

The EVs exhibited typical cup-shaped morphology and positive expression of canonical EV markers while being negative for the endoplasmic reticulum marker, confirming the successful isolation of high-purity CAR-T EVs. Proteomic profiling revealed that CAR-T EVs were enriched with 3,456 proteins, with 42% shared with their parental CAR-T cells. Notably, within immune-related pathways, 60% of the identified proteins overlapped, suggesting that CAR-T EVs retain a substantial portion of the immunomodulatory machinery of their parent cells.

Compared to CAR-T cells themselves, CAR-T EVs possess lower immunogenicity, an improved safety profile, and enhanced tissue-penetrating capacity (27). Consistent with previous reports (28), CAR-T EVs in this study were confirmed by Western blotting to express key immune effector molecules, including perforin, granzyme B, FasL, TRAIL, and NKG2D. CCK-8 assays indicated that CAR-T EVs exerted a concentration-dependent cytotoxic effect on HCC cell lines while showing no significant toxicity toward normal hepatocytes (THLE-2), aligning with the tumor-selective cytotoxicity reported for other CAR-EV platforms (29, 30).

Impaired NK cell function is a critical mechanism of immune evasion in HCC. Analysis of clinical samples revealed significant downregulation of NKG2D surface expression on peripheral blood NK cells from patients with HCC compared to healthy controls, consistent with the previously established miR-17-5p regulatory axis (14). Prior work from our group demonstrated that exosomal miR-17-5p, elevated in HCC tissues and cell lines, suppresses NK cell cytotoxicity by targeting RUNX1, a key transcription factor that binds to and activates the NKG2D promoter (14). The present study validated this axis *in vitro*, showing that lentiviral-mediated miR-17-5p overexpression in NK cells resulted in significant downregulation of both RUNX1 and NKG2D protein levels. Interestingly, Ding et al. reported that miR-17-5p can also regulate the expression of the NKG2D ligand ULBP2 in breast cancer by targeting MINK1 and modulating the JNK/c-Jun pathway (15), suggesting cell type– and context-dependent roles for miR-17-5p in modulating NK cell activity.

To achieve efficient delivery of miR-17-5p inhibitor, the Exo-Fect™ transfection reagent was employed for loading into CAR-T EVs, resulting in substantially higher loading efficiency compared to simple co-incubation. Agarose gel electrophoresis confirmed effective encapsulation of the inhibitor within the EVs, rather than surface adsorption. Delivery experiments demonstrated that EVs-Fect mediated efficient transfer to both HCC cells and NK cells. Current EV drug-loading strategies are broadly categorized into post-isolation methods (e.g., electroporation, lipofection, co-incubation) and pre-isolation methods (genetic or pharmacological modification of parent cells) (31). While electroporation offers high efficiency, it can compromise EV membrane integrity; in contrast, the lipid-based Exo-Fect method used in this study maintained EV structural integrity.

The core finding of this study indicates that miR-17-5p inhibitor–loaded CAR-T EVs effectively reverse the functional suppression of NK cells induced by miR-17-5p overexpression. EV-miR inhibitor restored the downregulated expression of NKG2D and RUNX1 in LV-miR NK cells to near-normal levels, whereas empty EVs had no such effect. Functional assays revealed significant impairment in the cytotoxic capacity and IFN-γ secretion of LV-miR NK cells against HCC cells compared to control NK cells, which were fully restored—and even enhanced—in the EV-miR inhibitor NK group. ELISPOT assays against Huh7 cells yielded consistent results. The zebrafish xenograft model provided *in vivo* validation, demonstrating that the EV-miR inhibitor NK group exhibited the most pronounced reduction in tumor burden and the greatest expansion of NK cell persistence.

Notably, IFN-γ secretion in the EV-miR inhibitor NK group not only recovered to baseline but surpassed that of the untreated NK control group, suggesting a potential “functionally augmented” state. This phenomenon may be explained by two non-mutually exclusive mechanisms. First, competitive inhibition of miR-17-5p may lead to a supra-physiological rebound in RUNX1 protein levels, thus potentiating NKG2D transcription and surface expression beyond normal levels, as RUNX1 serves as a key transcriptional activator of NKG2D (14). Second, the miR-17-5p inhibitor may relieve the repression of other potential target genes implicated in immune cell activation. For instance, miR-17-5p has been shown to target STAT3 in myeloid-derived suppressor cells and PTEN in other contexts (32), both of which could influence NK cell activity. Consequently, a multi-target regulatory effect could contribute to the observed functional enhancement. Additionally, while CAR-T EVs were removed by centrifugation prior to functional assays, trace amounts of residual effector molecules delivered during pre-treatment could exert a priming effect on NK cells. In summary, the primary mechanism underlying restored NK cell function is the specific blockade of the miR-17-5p/RUNX1/NKG2D axis, leading to reconstitution of tumor recognition and killing capacity.

The uptake efficiency of CAR-T EVs by NK cells is lower than that by tumor cells, attributable to several factors: (i) intrinsic membrane characteristics of NK cells as immune effectors (33); (ii) the specific molecular profile (e.g., adhesion molecules, ligands) on the surface of CAR-T EVs that governs cellular interactions (33, 34); and (iii) the influence of EV physicochemical properties, such as size distribution and surface charge, on cellular internalization (35).

Recent studies highlight the growing interest in engineered EVs for cancer immunotherapy. For example, Zhang et al. reported that EVs engineered to display CD19 and IL-12 significantly enhanced CAR-T cell IFN-γ secretion and cytotoxicity (36). Similarly, Hao et al. demonstrated that CAR-NKT–derived EVs could inhibit HCC progression by remodeling the immune microenvironment and boosting CD8^+^ T cell activity (37). These findings, along with the present work, underscore the potential of engineered EVs as a versatile immunotherapeutic platform.

This study provides the first evidence that CAR-T EVs can deliver miRNA inhibitors to reverse tumor-induced NK cell dysfunction. An efficient loading system was established and validated across *in vitro* and *in vivo* models, demonstrating functional reconstitution of NK cells and enhanced antitumor activity. However, several inherent limitations of the present study must be acknowledged. First, We acknowledged that direct, definitive validation of simultaneous occurrences of both direct tumor killing and NK cell function restoration within the same experimental system remains to be further investigated with dedicated assays. Second, the precise intravesicular localization of the miR-17-5p inhibitor (intraluminal vs. membrane-associated) requires validation via more precise techniques, such as cryo-electron microscopy. Lastly, the potential interactive relationship between the diverse bioactive molecules (e.g., perforin, granzyme B) carried by CAR-T EVs and the delivered miR-17-5p inhibitor requires further dissection. Addressing these questions will be crucial for advancing this combinatorial strategy toward clinical application.

Although the zebrafish model offers valuable insights into real-time tumor–immune cell interactions, its immune system differs from that of mammals. Future validation in immunocompetent murine HCC models is warranted. The observation of fluorescence area in zebrafish does not yet represent direct absolute quantification of the number of tumor cells or NK cells. Multiple confounding factors, including cell spreading, migration, aggregation, non-specific dye transfer, and inconsistent imaging conditions, may all interfere with the fluorescence area readings. Further in-depth verification at the single-cell counting level will be carried out in follow-up work. Notably, the zebrafish xenograft model has its inherent limitations that merit further discussion. First, compared to mammalian tumor models, the zebrafish immune system lacks the complexity of adaptive immunity (e.g., T cell differentiation and T cell-mediated immune regulation), and the cytokine secretion profile and immune cell composition in the TME differ significantly from those in mammals, complicating the reproduction of the human-specific immune regulatory network in tumor progression (38). Second, as a non-mammalian model, zebrafish exhibit significant differences in the biological distribution, clearance kinetics, and tissue penetration mechanisms of EVs compared to mammals, potentially leading to deviations in the prediction of *in vivo* efficacy in humans (39). Finally, the zebrafish model can only observe the short-term anti-tumor response during the development of the xenograft tumor, making it difficult to fully reflect the long-term anti-tumor persistence and metastasis inhibition effects of engineered CAR-T EVs *in vivo*. These limitations indicate that results obtained from the zebrafish model must be further validated in immunocompetent murine HCC models in the future to more accurately guide the clinical transformation of this therapy (40).

## Data Availability

The raw data supporting the conclusions of this article will be made available by the authors, without undue reservation.
