# Supplementary material for: Engineered CAR-T extracellular vesicles loaded with miR-17-5p inhibitor suppress hepatocellular carcinoma progression
Source: Front Immunol. 2026 Sep 16;17:1919449. doi: 10.3389/fimmu.2026.1919449 (PMC13624151; doi:10.3389/fimmu.2026.1919449)
Supplement: Supplementary file 1 [file Table1.docx]

**FIGURE s1. RTCA-based cytotoxicity detection of different treatments on hepatocellular carcinoma cells.**

The CI values of HCC cells in untreated NK, vehicle control, empty EVs and free miR-17-5p inhibitor groups were all higher than that in the control group, and there was no significant difference between the four groups.
